# *Pantoea bathycoeliae* sp. nov and *Sodalis* sp. are core gut microbiome symbionts of the two-spotted stink bug

**DOI:** 10.3389/fmicb.2023.1284397

**Published:** 2023-11-30

**Authors:** Arista Fourie, Stephanus N. Venter, Bernard Slippers, Gerda Fourie

**Affiliations:** Department of Biochemistry, Genetics and Microbiology, Forestry and Agricultural Biotechnology Institute (FABI), University of Pretoria, Pretoria, South Africa

**Keywords:** gut microbiome, stink bug, symbionts, *Pantoea*, *Sodalis*, gastric caeca

## Abstract

Stink bug species (*Pentatomoidea* superfamily) have developed an interdependence with obligate bacterial gut symbionts in specialized midgut crypts (M4 sub-region). Species of the *Enterobacteriaceae* family (predominantly *Pantoea*) are vertically transferred to their offspring and provide nutrients that cannot be obtained from plant sap food sources. However, the bacteria in the other gut compartments of stink bugs have rarely been investigated. The two-spotted stink bug, *Bathycoelia distincta*, is a serious pest of macadamias in South Africa. Nothing is currently known regarding its gut microbiome or how symbionts are transferred between insect generations. In this study, the consistency of *B. distincta* gut bacteria across geographic locations and life stages was determined with 16S rRNA metabarcoding, considering both the M4 and other gut compartments. A novel *Pantoea* species was found to be the primary M4 gut symbiont and is vertically transferred to the offspring. The other gut compartments had a low bacterial diversity and genera varied between stink bug populations but a *Sodalis* species was prominent in all populations. Sequence data of the M4 compartment were used to produce high-quality metagenome-assembled genomes (MAGs) for the *Pantoea* and *Sodalis* species. Functional analyses suggested a similar role in nutrient provision for the host, yet also unique metabolites produced by each species. The *Sodalis* sp. also had additional traits, such as secretion systems, that likely allowed it to establish itself in the host. The *Pantoea* species was described as *Pantoea bathycoeliae* sp. nov based on the rules of the SeqCode.

## 1 Introduction

Symbiotic associations between insects and bacteria have been established over long evolutionary time scales, and many of these associations are beneficial to the insect host (Cornwallis et al., 2023). Associations with gut symbionts have been investigated in numerous insects such as bees, termites, tsetse flies, beetles, aphids, and stink bugs (Engel and Moran, 2013; Gupta and Nair, 2020). These symbionts can contribute to nutrient provision and nutrient processing (Hansen and Moran, 2011; Moran, 2015), improving host immunity (Koch and Schmid-Hempel, 2011), overcoming plant host defense responses (Medina et al., 2018), protection against parasites (Oliver et al., 2009), or insecticide resistance (Kikuchi et al., 2012a).

One prominent long-standing insect-bacterial symbiosis is the gut symbionts of stink bugs (Duron and Noël, 2016; Hosokawa et al., 2016b). Stink bugs form part of the *Pentatomoidea* superfamily in the hemiptera order, consisting of more than 8,000 species (Rider et al., 2018). These insects have prominent associations with conserved extracellular symbionts that are maintained in midgut crypts, located in a subsection of their midgut (M4 region) (Salem et al., 2015; Hosokawa et al., 2016b). Usually, a single bacterial strain is maintained in high densities in this section. Physiological studies in a closely related family (*Alydidae*) suggest a constriction between the anterior part of the gut (M1–M3) and the M4 posterior section, allowing the obligate symbiont to enter the constricted section while food is bypassed from the M3 section to hemolymph and Malpighian tubules and then to the hind gut for excretion (Ohbayashi et al., 2015). In other species, once insects reach adulthood, the crypts become constricted and separated from the gut lumen and food can then again pass through this section (Oishi et al., 2019).

The obligate gut symbiont is generally transferred vertically from the mother to the offspring, through different methods in different species. Species in the *Pentatomidae* family perform an egg smearing on the surface of the eggs during oviposition (Calizotti and Panizzi, 2014), whereas the *Urostylididae* secrete a thick jelly that serves as a food source for the nymphs (Kaiwa et al., 2014). The *Parastrachiidae* secrete a thick mucus on the eggs just before hatching (Hosokawa et al., 2012), and the *Plataspidae* deposit small symbiont capsules along with the eggs (Hosokawa et al., 2005). The instars acquire the symbiont from the egg surface (or the symbiont capsules) after hatching and it is then maintained throughout their life cycle.

Many of the M4 gut symbionts are essential for the viability of their stink bug hosts. Experimental removal of the symbionts from *Nezara viridula* and *Plautia splendens* resulted in the mortality of the offspring (Tada et al., 2011; Hayashi et al., 2015). In other species, the effects were less severe but ranged from slower developmental rates to a reduction in body size or change in body color (Prado and Almeida, 2009; Kikuchi et al., 2012b; Hosokawa et al., 2013). In *Halyomorpha halys*, the detrimental effects, such as reduced fertility and viability, were only visible in the second generation (Taylor et al., 2014). Genome annotations and comparisons of numerous stink bug symbionts suggest that most of these species serve as nutritional symbionts, primarily contributing to the synthesis of essential amino acids and other vitamins and co-factors (Otero-Bravo et al., 2018; Kashkouli et al., 2021; Otero-Bravo and Sabree, 2021), but a role in uric acid recycling has also been suggested (Hosokawa et al., 2010). As stink bugs are xylem/phloem sap feeders, they obtain limited nutrients from their diet and additional amino acid and vitamin supplements are, therefore, essential (Douglas, 2006; Serteyn et al., 2020).

In the *Pentatomoidea* superfamily, each stink bug species shows a strong conservation of a specific bacterial species as an obligate symbiont. These bacterial species all form part of the *Enterobacteriaceae* family of *Gammaproteobacteria*, but different genera are prominent in the different *Pentatomoidea* families. For example, the largest stink bug family, *Pentatomidae*, predominantly contains *Pantoea* or *Erwinia* species (Duron and Noël, 2016), whereas all *Acanthosomatidae* contain one unique species, *Candidatus* Rosenkranzia clausaccus (Kikuchi et al., 2009), and the *Parastrachiidae* family maintains the species *Candidatus Benitsuchiphilus tojoi* (Mondal et al., 2020).

Limited studies have, however, focused on the bacterial species present in the other midgut compartments in stink bugs. A study of *N. viridula* identified two bacterial species in the M1–M3 section of the midgut (*Klebsiella pneumoniae* and *Enterococcus faecalis*), based on 16S PCR amplification and cloning (Hirose et al., 2006). Later studies identified both transient species, such as *Bacillus* spp., *Micrococcus* spp., *Streptomyces* spp., and *Staphylococcus* spp., and non-transient species including *Yokenella* spp. and *Enterococcus* spp (Medina et al., 2018). In *Antestiopsis thunbergia*, 16S rRNA PCR amplification and cloning from the midgut and ovaries identified *Sodalis*, *Spiroplasma*, and *Rickettsia* species throughout the gut and in other body tissues (Matsuura et al., 2014). In *Acrosternum arabicum*, *Gammaproteobacteria* and other taxa were detected in the M1 gut section (although less than in the M4 section) (Kashkouli et al., 2021). As most studies have focused on the M4 section, bacterial species in the rest of the digestive tract in stink bugs have likely been missed.

The two-spotted stink bug, *Bathycoelia distincta* (*Hemiptera*: *Pentatomidae*), is native to South Africa and is a serious pest in agricultural industries. It is one of the main contributors to nut losses in the macadamia industry in South Africa (est. 15.3 million USD annually) (Taylor et al., 2018), which is of great concern since South Africa is one of the largest exporters of this crop (SAMAC (South African Macadamia Association), 2021, Loss Factor Benchmark). The stink bug invasions in the orchards are predominantly controlled with insecticides, which is damaging to the environment and could lead to the development of insecticide resistance (Schoeman, 2019). The development of an integrated pest management approach, for example, targeting essential gut symbionts could improve control measures and reduce the use of insecticides (Gonella et al., 2020).

Nothing is currently known about the gut symbionts of *B. distincta* whether it also maintains an obligate symbiont in the M4 midgut section or how symbionts are transferred to the offspring. Additional facultative symbionts might also be hosted in other compartments of the stink bug gut. This study aimed to identify and compare the M4 midgut symbionts of *B. distincta* with those of other members of the *Pentatomidae* family, using a metabarcoding approach. In addition, the bacteria in the other gut compartments were also identified. To identify conserved symbionts, different geographic locations were included. To determine the potential transmission route of the symbionts and the microbes that are vertically transmitted, different developmental stages were considered. The functional roles of the primary symbionts in the host were predicted using metagenomic sequence data.

## 2 Materials and methods

### 2.1 Insect rearing and sample collection

A lab-reared population of *B. distincta* is being maintained at the Forestry and Agricultural Biotechnology Institute Biological Control Centre of the University of Pretoria, South Africa. This population was originally collected from an orchard in the Limpopo province (Limpopo 2) and has been maintained for nearly 4 years, at the time of the experiment. Rearing conditions entailed a constant temperature of 25°C–27°C and a 16:8 h day/night cycle. Insects were maintained in plastic containers with sufficient aeration and fed on *Zea mays* (maize) kernels on the cob.

Adult insects were collected from three orchards in the Limpopo province, namely Limpopo 1, Limpopo 2, and Limpopo 3, and from two orchards in Mpumalanga, namely Mpumalanga 1 and Mpumalanga 2, to determine if bacterial gut symbionts are conserved across geographic locations (Table 1). Insects were collected from knockdown sprays performed during routine scouting surveys. Different blocks were selected each week and 10 random trees were targeted, including both edges and trees deeper in the blocks. Collected insects were frozen at −20°C and stored in these conditions until dissections were performed. Between 7 and 11 insects were obtained from each of the orchards and from the lab-reared population for comparison of gut symbiont communities. Egg packages and different developmental stages were also collected from the lab-reared population to determine which bacterial species are likely vertically transmitted.

**Table 1:** Insect collections from the lab population, Limpopo (L) and Mpumalanga (MP) used for metabarcoding analyses and the number of ASVs observed in each.

Source	Province	GPS co-ordinates	No. of insects^a^	No. of ASVs (>1e−5 rel. abundance)	
				Rest gut	M4 section/instar
Lab population (adults)	N/A	N/A	11	486	142
Lab population (eggs)	N/A	N/A	30 (10 sets of 3)	—	260
Lab population (second instar)	N/A	N/A	30 (10 sets of 3)	—	181
Lab population (third–fifth instar)	N/A	N/A	1 each	—	225, 74, 149
Limpopo 1	L	−23.060287, 30.151149	7	438	95
Limpopo 2	L	−23.046212, 30.268774	11	503	86
Limpopo 3	L	−23.074688, 30.272448	11	475	100
Mpumalanga 1	MP	−25.807382, 30.998629	11 (8)	445	60
Mpumalanga 2	MP	−25.080501, 31.019021	9	442	61

a Number of insects collected differed from the final number of samples that were sequenced successfully. The number in brackets indicates the final number of samples used in the analysis.

### 2.2 DNA extraction for metabarcoding sequencing

**Figure 1:**
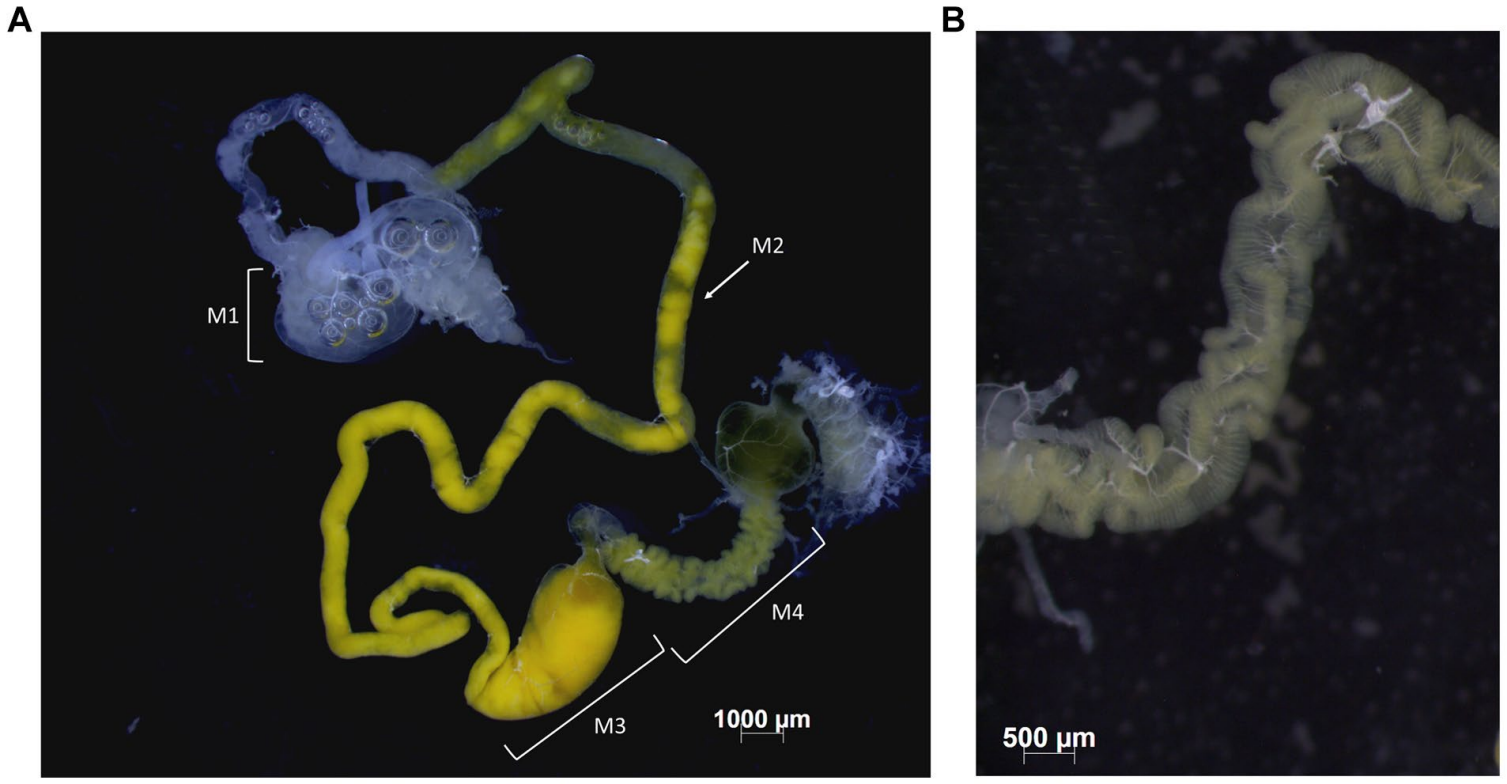
Midgut of *B. distincta*. **(A)** The entire midgut, indicating the different gut sections, from M1 to M4. **(B)** A close-up of the M4 midgut section where the midgut crypts, which contain a high abundance of the obligate symbiont, are clearly visible.

Adult insects were dissected under a dissection microscope to obtain the gut. Insects were first surface sterilized for 2 min each in 100% ethanol, then 2 × 70% ethanol, and rinsed in sterile water. The dorsal part of the insect was removed after which it was pinned onto filtered wax (Paraplast, Sigma-Aldrich, South Africa) in a Petri dish and dissected under phosphate-buffered saline solution with fine forceps. The M4 section of the midgut was separated from the rest, and both parts were rinsed in 70% ethanol and Sabax water and then stored at −20°C for DNA extractions (Figure 1). Due to the small size, all the M4 sections per orchard were combined and a bulk extraction was performed per orchard, but the rest of the gut was extracted individually for each insect. Single representatives of the other life stages (third, fourth, and fifth instar) were also included to track the dominant bacteria throughout the developmental stages. A similar dissection procedure was followed for these insects, but the gut was extracted as a whole. For the eggs and second instars, several samples were pooled to obtain 10 extractions of each stage. These pools either consisted of three egg packages each or three second instars each. Eggs were not surface sterilized, and second instars were sterilized by removing the head and legs and rinsing them in a similar manner as the adult insects.

DNA extractions for Illumina MiSeq 16S rRNA barcoding were performed for all samples (Table 1), using the QIAamp PowerFecal DNA Kit, and following the manufacturer’s instructions (Qiagen, Johannesburg, South Africa). DNA purity and concentrations were determined using a Thermo Scientific Nanodrop ND_100 spectrophotometer (Wilmington, DE, United States).

### 2.3 16S rRNA gene region amplification and sequencing

A 450 bp fragment of the V3/V4 section of the 16S ribosomal RNA region was amplified in all DNA samples using the Bakt_341F and Bakt_805R primer set (Klindworth et al., 2013), with Illumina adapter sequences added to the ends (forward: 5′ TCGTCGGCAGCGTCAGATGTGTATAAGAGACAG and reverse: 5′ GTCTCGTGGGCTCGGAGATGTGTATAAGAGACAG). PCR reactions were performed in triplicate per sample and then pooled before sequencing. PCR reactions were conducted in 25 μL reactions, consisting of ±50 ng DNA, 0.4 U Aq90 High fidelity DNA polymerase (Ampliqon, Denmark) and associated master mix, 0.5 μL of each primer (10 μM), 1–2 μL MgCl_2_ (25 mM stock), and Sabax water. The PCR conditions included 95°C for 3 min, 35 cycles of 95°C for 20 s, 51°C–54°C (ranged for optimization) for 30 s and 72°C for 30 s, and a final extension step at 72°C for 5 min. To determine PCR success, amplicons were analyzed on a 2% agarose gel via gel electrophoresis and visualized under UV light. Amplicons were purified with Agencourt AMPure XP beads (Beckman Coulter, CA, United States). Further library preparation included the addition of barcode sequences after which samples were combined for multiplex sequencing. Paired-end sequence data were generated using Illumina MiSeq technology. Library preparation and sequencing were performed at the Bioinformatics division, Agricultural Research Council, Pretoria, South Africa.

### 2.4 16S rRNA metabarcoding read processing and generation of amplicon sequence variants

Illumina MiSeq metabarcoding sequence reads were processed by removing primer sequences using Cutadapt (Martin, 2011). Reads were further filtered, trimmed, and denoised using DADA2 (Callahan et al., 2016) in R v. 4.0.3 (R Core Team, 2020), with parameters maxN = 0, maxEE = c(3,5), and truncQ = 2. Reads were then merged and chimeras were removed with DADA2 to obtain amplicon sequence variants (ASVs). Taxonomy was assigned using the SILVA database v.138,^1^ and ASVs assigned as chloroplast sequences were removed from the dataset. ASVs represented by very low numbers of reads (average relative abundance <1e−5) were removed from the dataset with phyloseq (McMurdie and Holmes, 2013) using R v. 4.0.3 (R Core Team, 2020) in RStudio (RStudio Team, 2019).

The ASV data were separated into two datasets for downstream analysis. The first dataset contained the M4 samples from all populations and the early developmental stages (eggs and second instars) from the lab-reared population (M4_LifeStages dataset). The second dataset contained the rest of the gut, excluding the M4, from all populations (Rest_gut dataset).

### 2.5 Analysis of gut microbiome based on 16S rRNA metabarcoding

#### 2.5.1 Gut community composition in different locations and different life stages

The abundance of different bacterial genera was investigated in the M4_LifeStages and the Rest_gut dataset of each population, based on ASVs with a relative abundance above 1e−5. ASVs were clustered according to genus classification using the Microbiome package (Lahti and Shetty, 2017). Bacteria were considered as core genera if they were present in more than 75% of the individuals with a relative abundance >1e−5 in all samples. For summarizing and visualizing the most abundant genera, all genera with a relative abundance >1% were used to construct a bar graph with ggplot2, for both datasets (Wickham, 2016). A bubble plot was also constructed for the Rest_gut dataset, based on relative abundance >1e−5, to illustrate the genera present in each individual using the ggplot2 package (McMurdie and Holmes, 2013). All analyses were done with RStudio (RStudio Team, 2019).

#### 2.5.2 Phylogenetic placement of conserved gut symbionts from all locations and different life stages

In the M4 gut section, a single bacterial species was expected, but various ASVs were obtained with the metabarcoding analysis. BLASTn analysis of the ASVs on the NCBI GenBank database (Sayers et al., 2019) either had similar matches to *Erwinia tasmaniensis* and *Pantoea septica* or matched to *Pantoea agglomerans*. The 16S rRNA gene region cannot properly differentiate between all *Pantoea* and *Erwinia* species (Brady et al., 2008); thus, a multilocus sequence typing (MLST) was used to determine the proper classification of the symbiont as an initial step. A DNA sample from the M4 section of a lab-reared insect, for which its 16S rRNA gene sequence was identical to the most abundant ASV, was used. PCR amplification was performed for three additional bacterial housekeeping genes (*atpD*, *rpoB*, *gyrB*) that can differentiate between *Pantoea* and *Erwinia* (Brady et al., 2008). The PCR reaction consisted of 50 ng DNA, 5 μL 5 × MyTaq^™^ buffer, 0.2 μL of 1 U MyTaq^™^ DNA polymerase (Bioline Ltd., United Kingdom), 0.5 μL of each primer (10 mM), and sterile Sabax water up to a total volume of 25 μL. PCR cycler and Sanger sequencing conditions were identical to Brady et al. (2008).

The consensus sequences obtained for each of the three gene regions were aligned with all *Pantoea* species for which sequence data for these genes were available in GenBank, as well symbionts in other stinkbugs classified as *Pantoea* sp. (Duron and Noël, 2016). *Kluyvera ascorbata* (strain ATCC 33433) and *Yokenella regensburgei* (strain ATCC 49455) were included as outgroups. Alignments were performed in MEGA v. 5 (Tamura et al., 2011), using Muscle. Sequences were concatenated and a phylogenetic tree was constructed, based on a maximum likelihood analysis, using the GTRGAMMA setting and 1,000 bootstrap replications in RaxML v. 8.2.11 (Stamatakis, 2014).

To determine the phylogenetic placement and clustering of the 10 most abundant *Pantoea* ASVs (metabarcoding data) from the M4 gut sections and the different life stages, a reduced phylogenetic tree was constructed, including the 16S rRNA sequences of closely related *Pantoea* species and stink bug symbionts. The symbiont of a related *Bathycoelia* species, *Bathycoelia indica*, was specifically included to test for any relatedness between their symbionts. *Pseudomonas syringae* (strain ICMP 3023) was used as an outgroup for the tree. Phylogenetic relationships were determined, as described above. This is the only step where the third–fifth instar ASVs were included in analyses since these samples were represented by single individuals.

The other prominent species, observed in the Rest_gut dataset of all geographic locations and in the different developmental stages, was a *Sodalis* species. To use the full-length 16S rRNA of this species for more accurate phylogenetic placement, the sequence was obtained from the metagenome-assembled genome (MAG, see section 2.7) and the five most abundant *Sodalis* ASVs were aligned to this sequence to determine similarity. Each ASV differed in only one nucleotide (thus 99.77% similarity) from the metagenomic 16S rRNA sequence, and hence, this sequence was used to represent the phylogenetic placement of the *Sodalis* species. Additional *Sodalis* isolates collected from other insects, including various stink bug species, were included (available on NCBI, GenBank) (Sayers et al., 2019) and alignments and phylogenetic analyses were performed similar to the methods described above.

#### 2.5.3 Diversity analysis of bacterial communities in the Rest_gut dataset across geographic locations

The richness and evenness (alpha diversity) of the bacterial communities present in the rest of the gut of each individual were determined using Shannon’s diversity index (richness and evenness with more weight on richness), Simpson’s index (richness and evenness with more weight on evenness) and Chao1 diversity index (richness). The absolute read counts for each ASV were used, excluding ASVs with less than <1e−5 relative abundance. Significant differences in the alpha diversity of the different populations were tested with Kruskal–Wallis, followed by a Wilcoxon rank sum test to identify the populations that differed significantly. The Benjamini and Hochberg method was used to adjust *p*-values for multiple comparisons.

To determine if any of the populations had distinct microbial communities, a β-diversity analysis was performed with the Rest_gut data, using the relative abundance of the ASVs (abundance >1e−5). Distances were calculated based on Jaccard distance (which does not consider the abundance of different ASVs) and Bray–Curtis dissimilarity (which considers composition and abundance of ASVs). The homogeneity of data dispersion in each group was confirmed, using betadisper, followed by a permutational multivariate analysis of variance (permanova) using adonis in the vegan package (Oksanen et al., 2020). To determine the populations that were significantly different from one another, a pairwise comparison was performed using the pairwiseAdonis package and *p*-values were adjusted with the Benjamini and Hochberg method (Martinez, 2020). In addition, the distances were used to construct an NMDS ordination plot to visualize the clustering of individuals from all populations. All analyses were performed with R v. 4.0.3 (R Core Team, 2020) in RStudio (RStudio Team, 2019).

### 2.6 Metagenome sequence analysis of gut symbionts

#### 2.6.1 DNA extraction and sequencing of a metagenome

Given the potentially unique species identified through the MLST approach described above, DNA extractions for metagenome sequencing were performed for the M4 section. The M4 gut section of three female insects from the lab-reared population were combined. DNA was extracted using the Macherey-Nagel NucleoSpin Tissue kit (Düren, Germany) and an RNase A treatment was included in the protocol. DNA purity was determined with a Thermo Scientific Nanodrop ND_100 spectrophotometer (Wilmington, DE, United States), and concentrations were determined with a Qubit^®^ 2.0 Fluorometer (ThermoFisher Scientific). The DNA that passed the purity filters of 260/280 ratio >1.7 and minimum concentration >20 ng/μL was sent for Illumina TruSeq PCR-free sequencing at Macrogen, Seoul, Republic of Korea. The DNA libraries were constructed using the TruSeq Nano DNA kit (Illumina, CA, United States). DNA was randomly fragmented, 5′ and 3′ adapters were ligated, and a 350 bp insert size was used for paired-end sequencing.

#### 2.6.2 Metagenome assembly and binning

The sequence reads obtained from Illumina TruSeq were quality checked using fastqc (Andrews, 2010) and trimmed using Trimmomatic v. 0.38 (Bolger et al., 2014), based on a phred quality score above 30, minimum length 100 bp, using a sliding window of 4:30 and an average read quality of 20. The trimmed reads were analyzed with fastqc again to ensure all adapters were removed. To remove host-contaminating DNA, the reads were mapped to an in-house available draft genome sequence of the *B. distincta* host, using bowtie2 (Langmead and Salzberg, 2012). All unmapped reads were collected for metagenome assembly.

Metagenomic reads were assembled using the MetaSpades mode in Spades v. 3.15.3 (Nurk et al., 2017). Quality filtered reads were mapped back to the assembly, using bowtie2 (Langmead and Salzberg, 2012), to determine the assembly success rate and for contig binning. Contigs were grouped into bins using MaxBin 2.0 (Wu et al., 2015) and MetaBAT 2 (Kang et al., 2019), respectively, after which bins were dereplicated using MiGA (Rodriguez-R et al., 2018). Phylogenetic identity, genome completeness, and contamination of the bins were determined with the MiGA webserver^2^. Two bins with high genome completeness were obtained and were identified as *Pantoea* and *Sodalis*, respectively. To identify any contaminant contigs in the bins (MAGs), taxonomic classification of contigs was performed using the Contig Annotation Tool (CAT) (von Meijenfeldt et al., 2019). Genome completeness of the MAGs was confirmed with BUSCO (Manni et al., 2021), using the core *Pseudomonodota* (previous *Proteobacteria*) protein dataset as a reference. The closest related genomes in the Genome Taxonomy Database (GTDB) were identified for both genomes, using GTDB-Tk-v1.7.0 (Chaumeil et al., 2019) on the Kbase server (Arkin et al., 2018).

#### 2.6.3 Genome annotation and functional predictions

Gene prediction and functional annotation were performed for the *Pantoea* and *Sodalis* MAGs, using Prokka (Seemann, 2014), including the –compliant and –rfam options and providing the appropriate genus as a reference for annotation. Pseudogenes were predicted with Pseudofinder (Syberg-Olsen et al., 2022), which compares proteins both to a non-redundant protein database and to its closest reference genome to identify genes distinctly smaller or larger than its average ortholog, containing frameshifts, early stop codons, or no stop codons as parameters for pseudogene prediction. The genomes *Pantoea septica* (GCF_902386985.1) and *Sodalis glossinidius* (GCF_000010085.1) were used as reference genomes for each species, respectively, and additional parameters that were modified included --length_pseudo 0.5, --shared_hits 0.7, --intergenic_length 100, --intergenic_threshold 0.9, and --hitcap 30. Specific functional groups, such as carbohydrate-active enzymes (CAZymes) and peptidases, were further summarized using dbCAN2 (Zhang et al., 2018) (only considering features detected by both HMMER and Diamond) and BLASTp to the MEROPS database (E-value <1e−6, >40% ID, >40% query coverage) (Rawlings et al., 2013), respectively. KEGG functional annotations were added to the proteins, using KofamKOALA (Aramaki et al., 2019), and metabolic pathways were identified with KEGG Mapper (Kanehisa and Sato, 2020). Secondary metabolite gene clusters were predicted with antiSMASH (Blin et al., 2021), and secretion systems were predicted with MacSyFinder (Abby et al., 2014).

### 2.7 Phylogenomic placement and genome comparisons of *Pantoea* symbiont within *Pantoea* genus

The MAG of the primary symbiont, *Pantoea* sp., was further investigated to determine its relatedness to other *Pantoea* species, both free-living and other stink bug symbionts. Publicly available genomes for all well-defined free-living *Pantoea* species, as well as symbionts from other *Pentatomidae* stink bugs, were obtained from NCBI (Supplementary Table S1). The average nucleotide identity (ANI) between the *Pantoea* symbiont and other *Pantoea* genomes was determined using MASH (Ondov et al., 2016).

The similarity in gene content between the genomes was determined by identifying shared and unique orthologs, using OrthoFinder v2.2.6 (Emms and Kelly, 2019) with standard parameters. This could identify any genes unique to the *Pantoea* symbiont. The OrthoFinder output was also used to identify the core set of single-copy genes that were used for phylogenomic tree reconstruction. The *Pseudomonas syringae* strain BIM B-268 (GCF_016694755.2) was used as an outgroup to root the phylogenetic tree and was thus included in the OrthoFinder analysis of single-copy core genes. A total of 328 single-copy genes were identified, and each was individually aligned with MAFFT v7.407 (Katoh and Standley, 2013); poorly aligned regions were removed with trimAl (Capella-Gutiérrez et al., 2009), after which all genes were concatenated with FASconCAT-G (Kück and Longo, 2014). A phylogenomic tree was constructed with RaxML v8.2.12 (Stamatakis, 2014) with the concatenated gene alignment, as well as partition data for each gene as generated by FASconCAT-G, using the GAMMA model of rate heterogeneity. One thousand bootstrap replicates were performed to determine branch support values.

## 3 Results

### 3.1 16S rRNA metabarcoding read processing and generation of amplicon sequence variants

The Illumina MiSeq data obtained for the metabarcoding ranged from 56,000 to 190,000 raw paired reads per sample, with an average of 121,000 reads. After quality filtering and chimera removal, the reads ranged from 166 to 140,000, with an average of 45,500 read pairs per sample. Three of the samples had very few reads retained after filtering and chimera removal (all three from Mpumalanga 1), and these were excluded from further analyses.

A total of 1,447 ASVs (excluding chloroplast sequences) were obtained in the full dataset from the DADA2 analyses. After the removal of ASVs with a relative abundance <1e−5, 331 of these ASVs were retained in the M4_LifeStages dataset and 603 in the Rest_gut dataset. Of these filtered ASVs, individuals each contained between 61 and 239 ASVs (avg. 119) in the M4_LifeStages dataset (Supplementary Table S2) and between 20–364 ASVs (avg. 168) in the Rest_gut dataset (Supplementary Table S3), respectively.

### 3.2 Analysis of gut microbiome based on 16S rRNA metabarcoding

#### 3.2.1 Gut community composition in different locations and different life stages

**Figure 2:**
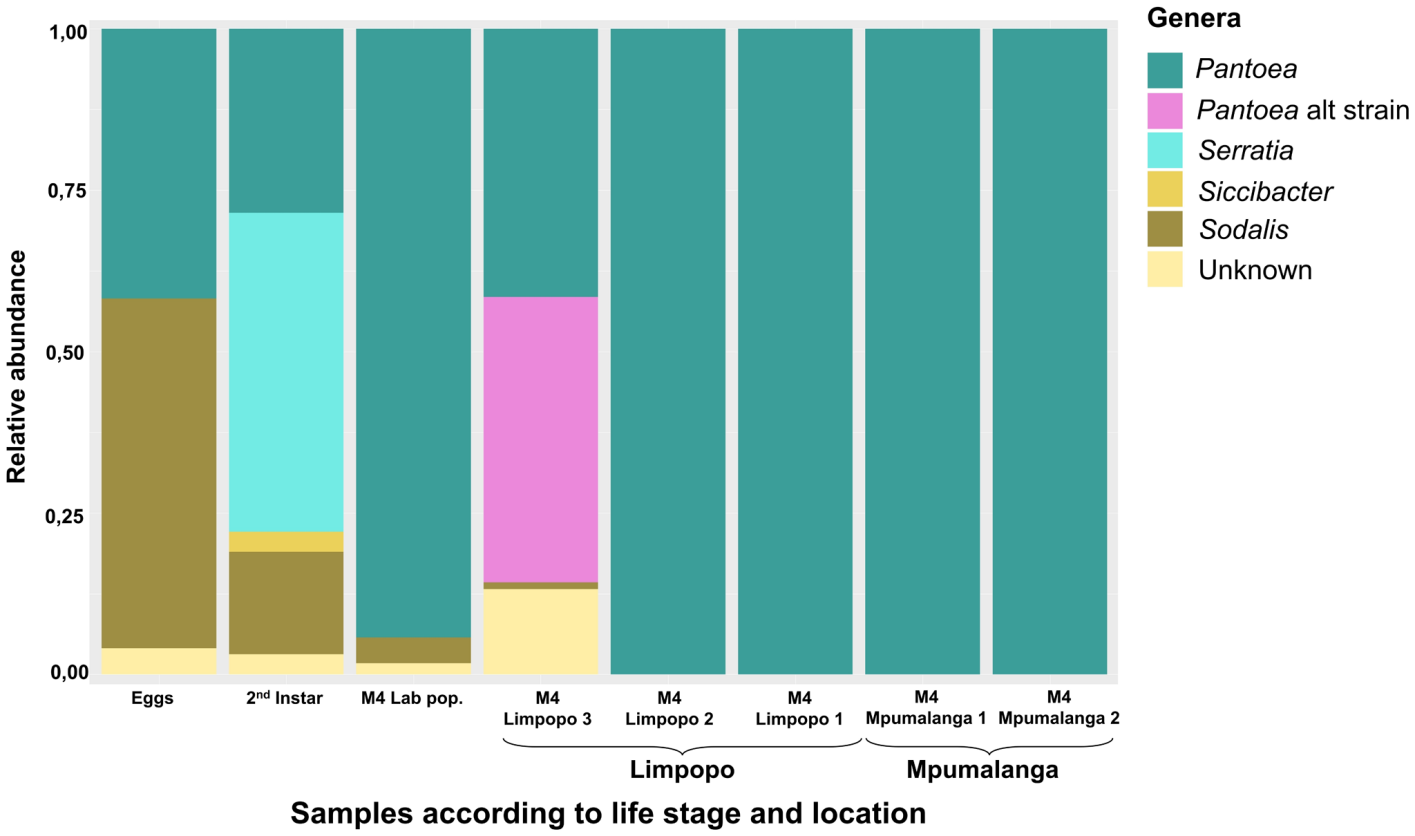
Relative abundance of the most prominent bacterial genera (>1%) present in the M4 midgut section of stink bugs from different locations as well as in the early developmental stages of the lab-reared population. The samples were bulked and represent a number of individuals (8–11).

The bacteria present at the different developmental stages of the lab-reared population and those in the M4 gut section of all populations were compared to identify genera maintained from birth and conserved across locations. *Sodalis*, *Serratia*, *Siccibacter*, and *Pantoea* were present in the different life stages, of which only *Sodalis* and *Pantoea* were consistent in all stages (Figure 2). For example, the most abundant *Serratia* ASV (ASV7) was only present in the eggs, 2nd and 5th instar with a high abundance in the 2nd instar. The M4 section predominantly contained one *Pantoea* ASV (ASV1), but an additional *Pantoea* strain/species was present in the Limpopo 3 population (Figure 2). Additional ASVs, classified as *Pantoea*, were 99.7%–99.9% similar to ASV1 (ASV4, ASV9, ASV10, ASV13, ASV18). The alternative *Pantoea* strain was represented by four ASVs (ASV16, ASV26, ASV28, ASV45) that were predominantly present in the Limpopo 3 population. These ASVs were also 99.8% similar to one another but 98.5% similar to ASV1, suggesting a distinct strain.

Both *Sodalis* and *Pantoea* were present on the eggs and throughout the life stages, suggesting a vertical transfer of these bacteria from parents to offspring. Newly hatched instars also displayed a probing behavior on the egg surfaces after hatching (Supplementary material S1), where they likely obtained the bacteria. However, *Sodalis* could also have been transferred transovarially as it is known to be an endocellular symbiont.

**Figure 3:**
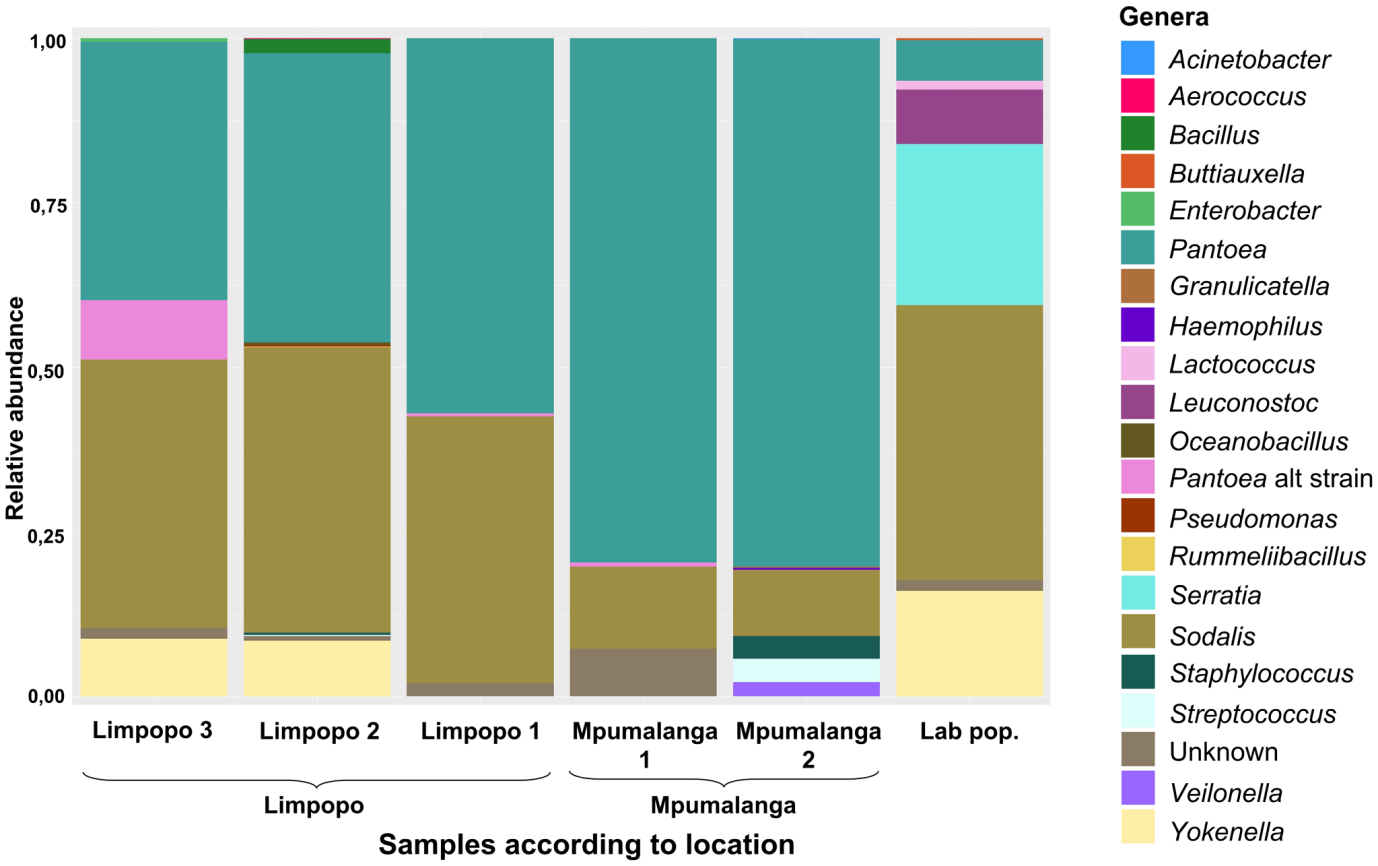
Relative abundance of the most prominent bacterial genera (>1%) present in the rest of the gut (excluding the M4 region), according to geographic location. Each location is represented by 7–11 samples.

In the Rest_gut dataset, the Enterobacterales order (*Pseudomonodota* phylum—previously known as the *Proteobacteria*) was most abundant, including the genera *Sodalis*, *Pantoea*, *Yokenella*, and *Serratia* (Figure 3). Less abundant ASVs (yet still >1%) were assigned to the *Bacillota* phylum (previously *Firmicutes*), including the genera *Aerococcus*, *Bacillus*, *Granulicatella*, *Leuconostoc*, *Lactococcus*, *Oceanobacillus*, *Rummeliibacillus*, *Staphylococcus*, *Streptococcus*, and *Veilonella* and other *gammaproteobacteria* of the genera *Acinetobacer*, *Buttiauxella*, *Enterobacter*, *Haemophilus,* and *Pseudomonas*. All genera observed in the populations (RA >1e−5) are represented in Supplementary Figure S1.

The core genera (present in >75% of the samples and RA >1e−5) were *Pantoea*, *Serratia*, and *Sodalis*, while others were in lower abundance (or only abundant in some populations) such as *Atlantibacter*, *Buttiauxella*, *Leuconostoc*, *Siccibacter*, *Staphylococcus*, and *Streptococcus*. *Sodalis* was primarily represented by five abundant ASVs, all with 99.7% similarity. The species was present in all populations (although at variable RA in individuals) and was in all individuals of the lab and Limpopo populations but only in 50% of the individuals of the Mpumalanga populations (Supplementary Figure S1). *Pantoea*, however, was present in all individuals, which confirms this as the most conserved symbiont. This species was represented by 10 abundant ASVs. ASV4, ASV9, and ASV15 were all 99.8% similar to ASV1. Although in low abundance, ASV4 and ASV15 were present in most individuals, while ASV9 was abundant in some but absent from many individuals. The additional strain/species of *Pantoea* (such as ASV16) was observed in the Limpopo 3 population although this was predominantly in one individual with very low abundances in other individuals (Figure 3; Supplementary Figure S1). *Serratia* occurred in low abundance in most individuals but was more abundant in some individuals in the lab population. Sequence similarity between ASVs of *Serratia* were all above 99.2%.

#### 3.2.2 Phylogenetic placement of conserved gut symbionts from all locations and different life stages

**Figure 4:**
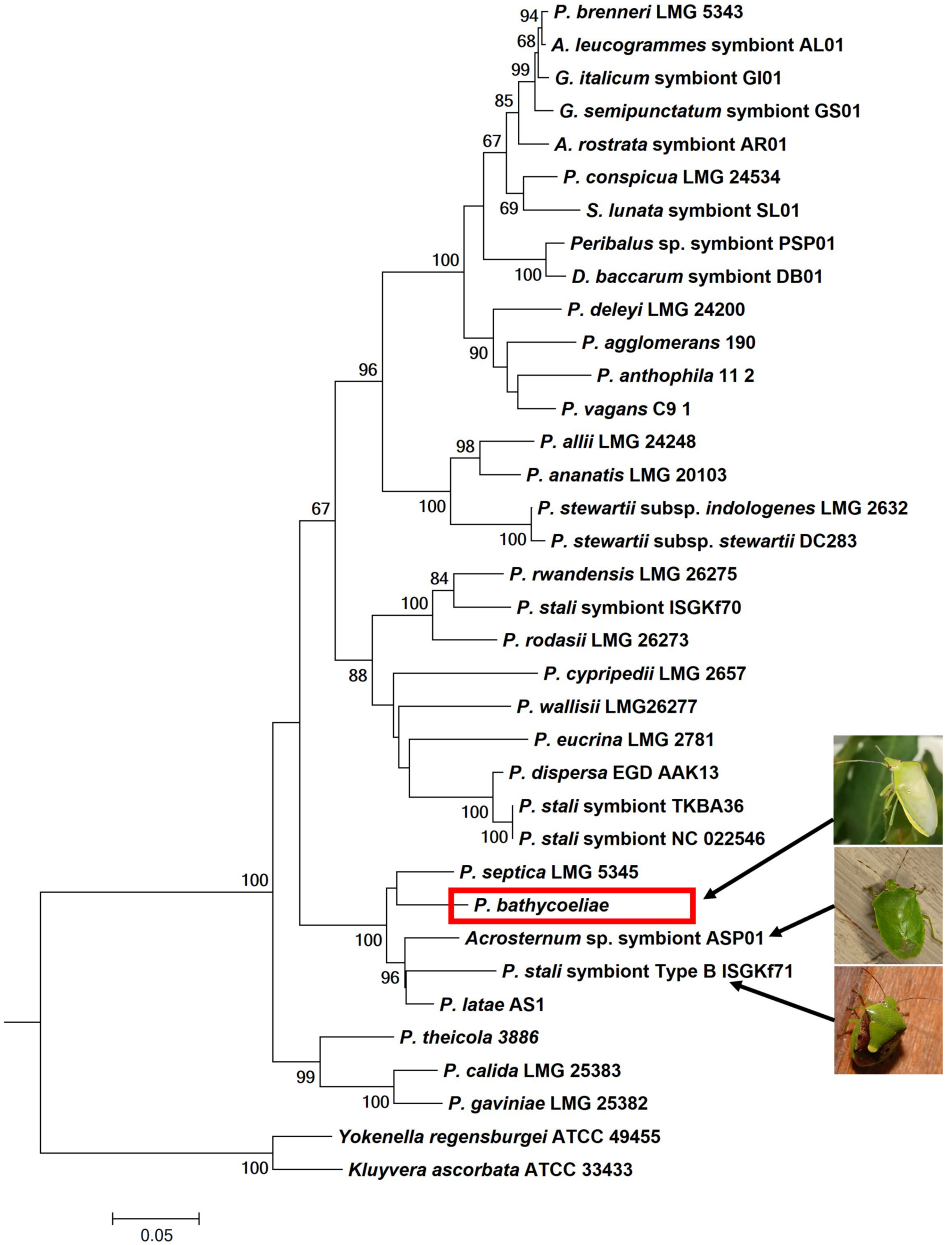
Phylogenetic placement of the essential gut symbiont of *B. distincta* (red block), based on the combined sequence of the *atpD*, *rpoB,* and *gyrB* genes. This tree represents the most likely tree, based on maximum likelihood analyses, and branch support based on 1,000 bootstrap replications is indicated on the branches. This illustrates the most closely related free-living species is *Pantoea septica* and the closest stink bug symbionts are those from *Acrosternum* sp. and *Plautia stali* (indicated by the arrows and pictures).

In the phylogenetic tree of the combined gene regions (*atpD*, *rpoB*, and *gyrB*), the conserved *B. distincta* symbiont grouped closest to the free-living *Pantoea* species, *P. septica*, and was also closely related to “*P. latae*” and the gut symbionts of an *Acrosternum* stink bug species and the *Plautia stali* symbiont B (Figure 4). This phylogenetic tree supports that the *B. distincta Pantoea* symbiont is a novel species and is from here on referred to as *Pantoea bathycoeliae*, for ease of reference. A formal description of the species follows at the end of this section based on the MAG obtained from the metagenomic data of the M4 gut section.

**Figure 5:**
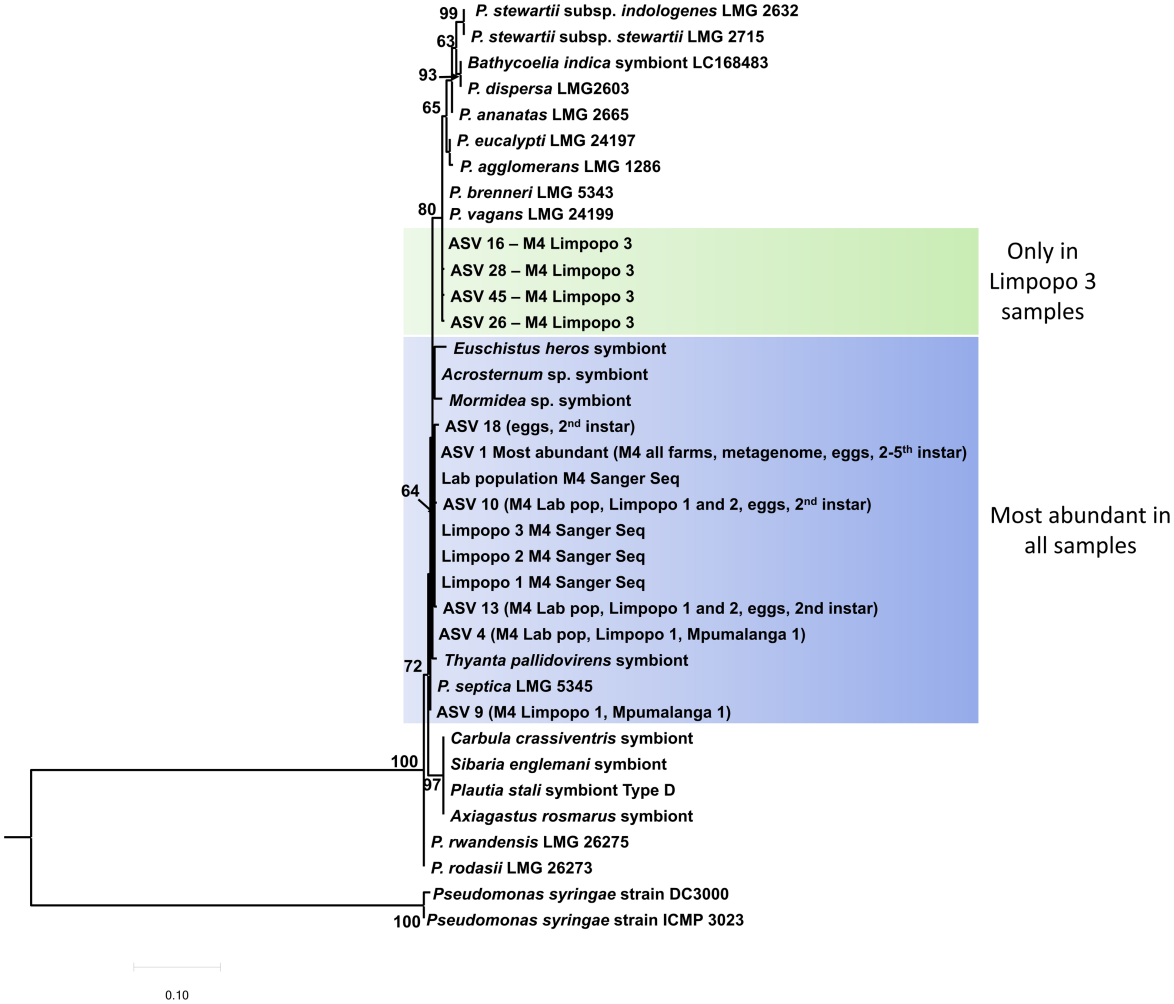
Maximum likelihood tree of the 10 most abundant *Pantoea* ASVs (16S rRNA metabarcoding) present in the M4 gut sections from all locations and the different developmental stages. The most abundant ASV (ASV1) was present in the M4 section from all locations and in all developmental stages (eggs, second–fifth instar). An additional strain/species was present in the Limpopo 3 samples, which grouped with *P. vagans* and *P. brenneri*. Branch support values >60% are indicated at the branches, based on 1,000 bootstrap replicates. *Pseudomonas syringae* was selected as outgroup to root the tree.

The most abundant *Pantoea* ASVs, identified in the 16S rRNA metabarcodes, could be classified in a 16S rRNA phylogenetic tree (Figure 5). Six of the ASVs grouped close to *Pantoea septica* and the symbionts from four stink bug species. These six ASVs were 99.6%–99.9% similar to one another and are likely variations introduced due to minor sequence errors, or could be different strains of the same species. The additional *Pantoea* strain/variant that was present in the M4 section and rest of the gut in the Limpopo 3 samples (represented by four ASVs) was clearly a distinct species from *P. bathycoeliae* and grouped with *Pantoea vagans* and *Pantoea brenneri*. The *Pantoea* symbiont from the closely related stink bug species, *B. indica*, did not show any close relation to the *B. distincta* symbiont.

**Figure 6:**
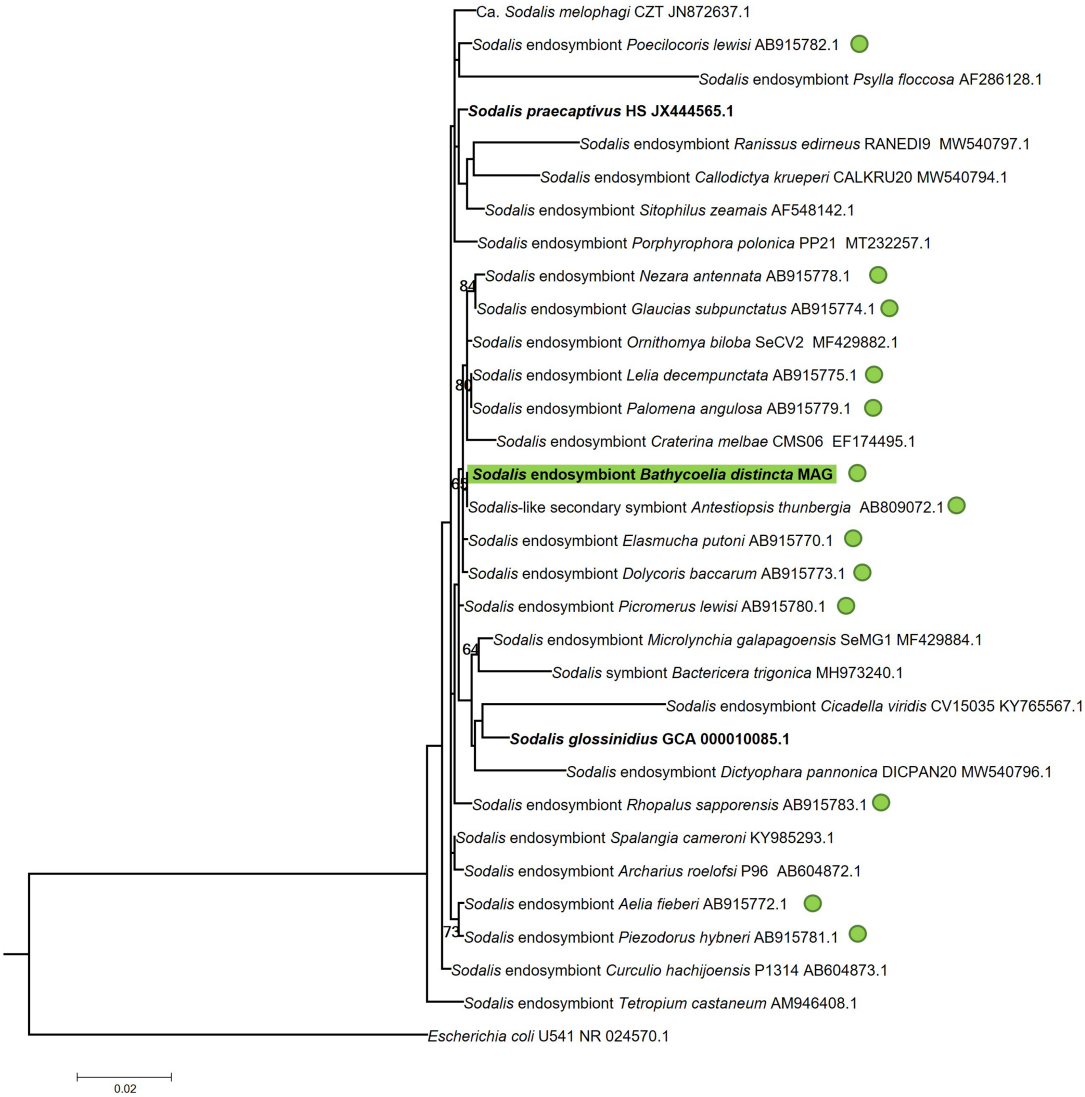
Maximum likelihood tree of the 16S rRNA gene region of the *Sodalis* symbiont identified in the *B. distincta* gut in relation to *Sodalis* strains identified in other stink bugs and other insects. The *B. distincta* symbiont is highlighted in green, and all other stink bug species are indicated with green dots. *Escherichia coli* was selected as outgroup to root the tree. Branch support values >60% are indicated, based on 1,000 bootstrap replicates.

In the 16S rRNA phylogenetic tree of the *Sodalis* symbiont, most of the strains could not be properly differentiated by this gene region or had low branch support values (Figure 6). The *Sodalis* isolate from *B. distincta* was nearly identical to a strain previously collected from *Antestiopsis thunbergia* (*Hemiptera*: *Pentatomidae*), a pest of coffee plants in Africa. This is likely the same bacterial species occurring in different stink bug species but the availability of a full genome would provide more clarification.

#### 3.2.3 Diversity analysis of bacterial communities in Rest_gut dataset across geographic locations

The alpha diversity of each population (Rest_gut dataset) was compared to determine if any populations differ in diversity. The Mpumalanga populations had a significantly lower diversity compared to each of the Limpopo populations and the lab population, based on multiple diversity indices (Chao1, Shannon and Simpson; Pairwise Wilcoxon Rank Sum Tests; Supplementary Figure S2). Interestingly, the lab population had higher Shannon’s and Simpson’s diversity indices than its original source population, Limpopo 2, but they were not more diverse than the other Limpopo populations.

There was a significant difference in community composition between the populations, based on both Jaccard (*R*^2^ = 0.17552) and Bray–Curtis distances (*R*^2^ = 0.1859, *p* < 0.05). This was mainly attributed to the difference between the lab population and the Mpumalanga populations (pairwise comparison *p* = 0.015, Supplementary Table S4). Overall, the Mpumalanga populations (Mpumalanga 1 and Mpumalanga 2) were relatively distinct from the lab and Limpopo populations, as seen in the NMDS plot (Supplementary Figure S3). This is likely due to the lower species diversity in these populations.

### 3.3 Metagenome-assembled genomes of two core gut symbionts

#### 3.3.1 Genome assembly and binning

Nearly 207.3 million reads were retained from Illumina TruSeq sequencing of the metagenome, after quality filtering and removal of host reads. The contig assembly, with MetaSpades, resulted in 354,262 contigs with an N50 value of 2,687 bp. The assembly rate, based on the percentage of reads that mapped back to the assembly, was 88.14%. The contig binning resulted in four bins of which two where high-quality bins with low contamination (<2%) and high completeness scores (>95%). Bin1 was classified as a *Pantoea* species and bin2 as a *Sodalis* species based on CheckM analysis. Comparison to the GTDB did not find any closely related species to the *Pantoea* genome, and the closest match to the *Sodalis* genome (98.7% ANI) was *Sodalis praecaptivus*. Alignment of the 16S rRNA sequences from the two metagenome-assembled genomes (MAGs) to the ASVs from the metabarcoding data confirmed these species to be identical to the most abundant *Pantoea* (*P. bathycoeliae*) and *Sodalis* ASVs (ASV1 and ASV2, respectively).

The *Pantoea bathycoeliae* MAG had a 100% completeness and 1.9% contamination score, based on CheckM, and a 97% completeness based on BUSCO analysis. The genome consisted of 127 contigs with a total length of 3.16 Mbp, an N50 of 70.5 Kbp, and a GC content of 54.85%. The genome coverage had an average sequence depth of 3,333. The number of predicted genes was 4,180, including 4,058 protein-coding genes, 48 tRNA, and 71 other RNA transcripts. The Pseudofinder analysis joined 460 ORFs as fragmented pseudogenes, resulting in a final prediction of 1,747 pseudogenes and 2,128 intact protein-coding genes.

The *Sodalis* sp. MAG was 95.3% complete with 0.9% contamination (CheckM) and a 98.4% BUSCO completeness score. The genome consisted of 453 contigs with a total length of 3.57 Mbp, an N50 of 15.6 Kbp, and a 56.7% GC content. The genome coverage had an average sequence depth of 686. A total of 3,822 genes were predicted, including 3,688 protein-coding genes, 45 tRNA genes, and 88 other RNA transcripts. The Pseudofinder analysis predicted the joining of 94 ORFs as fragmented pseudogenes, resulting in a final set of 753 pseudogenes and 2,916 intact protein-coding genes. The genome assembly JAUHTO000000000 is available under the GenBank BioProject PRJNA987703 and BioSample SAMN36080331.

#### 3.3.2 Functional annotations of *Pantoea* and *Sodalis* MAGs

**Figure 7:**
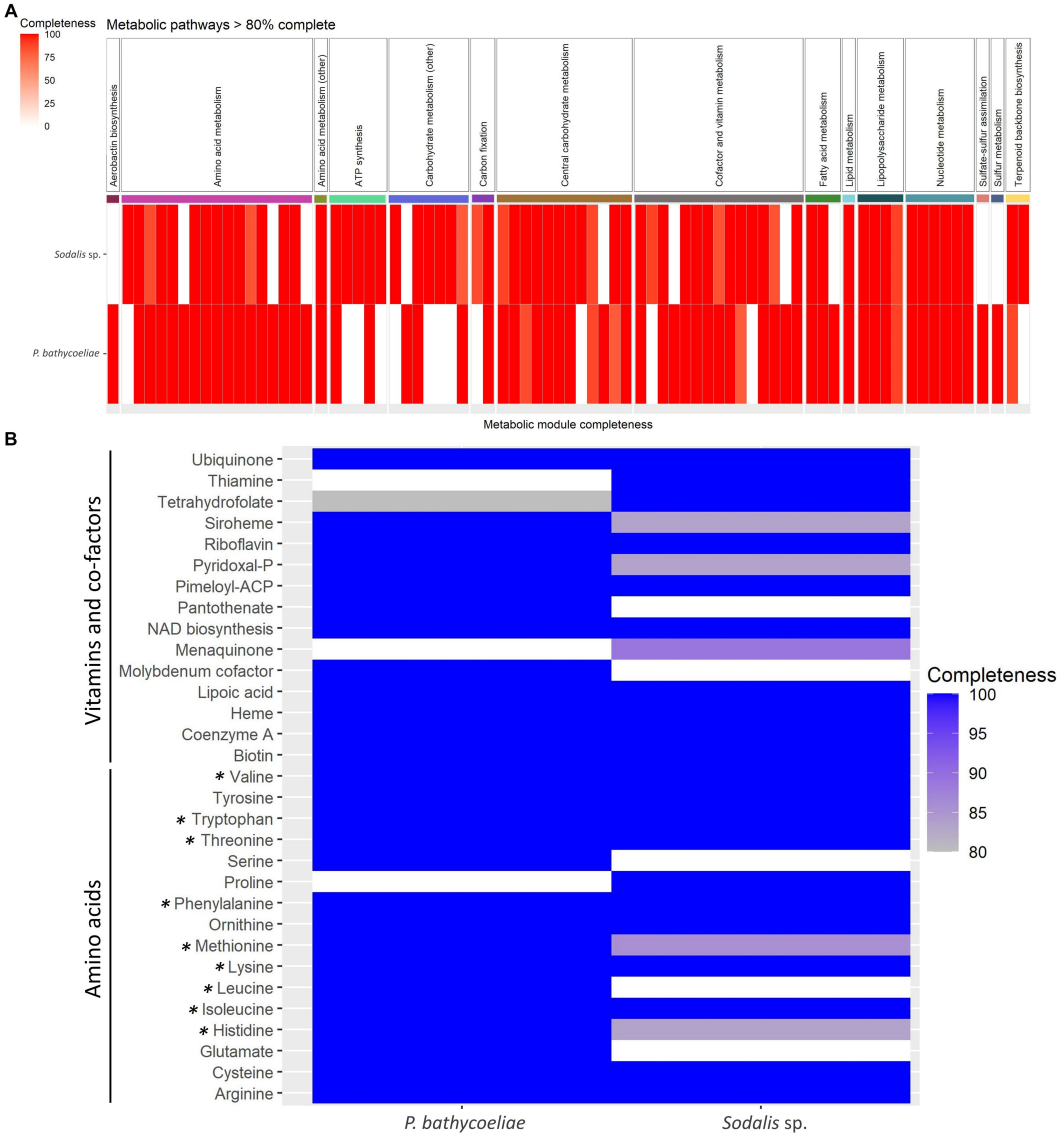
**(A)** All metabolic models detected in the genomes of the *Pantoea* and *Sodalis* symbiont of *B. distincta* with >80% completeness. **(B)** Detailed summary of amino acids, vitamins, and co-factors modules. All essential amino acids are labeled with an asterisk.

The metabolic pathway modules predicted in the genomes of the *P. bathycoeliae* and *Sodalis* sp. MAGs indicated a high similarity in their metabolic potential, with primary metabolic modules (>80% complete) related to carbon fixation, carbohydrate metabolism, nucleotide metabolism, ATP synthesis, and biosynthesis of amino acids, vitamins, and other co-factors (Figure 7A). Complete or near-complete (one enzyme missing) modules were present for 15 and 13 amino acids in the *P. bathycoeliae* and *Sodalis* sp. MAGs, respectively. All nine essential amino acids had complete pathway modules in *P. bathycoeliae*. In the *Sodalis* sp., the leucine, methionine, and histidine modules had one enzyme missing (LeuA, MetA/X, and HisD/HIS4) (Figure 7B) of which the LeuA and HisD genes were predicted pseudogenes, suggesting they are no longer functional. In addition, the *Sodalis* sp. had a complete pathway for proline, while some genes were not functional in *P. bathycoeliae*. *P. bathycoeliae* and *Sodalis* each had complete (or near-complete) modules for 13 vitamins and co-factors. The vitamins and co-factors were highly similar between the two species: *P. bathycoeliae* uniquely encoded for pantothenate and molybdenum metabolism and *Sodalis* sp. for menaquinone and thiamine.

Additional metabolic modules of importance, that were present in both species, included lipopolysaccharides, and the terpenoid C5 isoprenoid biosynthesis, while only *P. bathycoeliae* had a complete pathway for aerobactin production (iron chelating agent) and for sulfur metabolism and sulfate–sulfur assimilation. Sulfur assimilation, through sulfate reduction, contributes to the incorporation of sulfur into methionine and cysteine amino acids (Hansen and Moran, 2014).

*Pantoea bathycoeliae* encoded for 30 carbohydrate-active enzymes (CAZymes), involved in the synthesis or breakdown of carbohydrates. These primarily consisted of glycoside hydrolases and glycosyl transferases (Supplementary Table S5A). A total of 78 peptidases and four inhibitors were encoded, of which metallopeptidases and serine peptidases were most abundant (Supplementary Table S5B). Of particular interest were families with high abundance in the genome, including C26 (gamma-glutamyl hydrolase), M20A (glutamate carboxypeptidase), and M23B (beta-lytic metallopeptidase). The *Sodalis* sp. encoded for 44 CAZymes and had more GH and GT families than *P. bathycoeliae*. The highest abundance was six peptidoglycan lyases GH23, and it had more glycosyltransferases (GT2, GT4, GT9) involved in the breakdown of sugars and lipopolysaccharides. The species encoded for 116 peptidases and four inhibitors but encoded for more cysteine peptidases than *P. bathycoeliae* and had less proteins of the M20A family but more of the M38 (isoaspartyl dipeptidase) and S33 (prolyl aminopeptidase) families.

Only one known, complete secondary metabolite cluster was identified using antiSMASH, a terpenoid biosynthetic gene cluster encoding for carotenoid synthesis in the *P. bathycoeliae* genome. The cluster had 78% similarity to a carotenoid cluster in a related *Erwiniaceae* species, *Kalamiella piersonii*, and 50% similarity to clusters in *Pantoea agglomerans* strains. All the genes from this pathway matched the carotenoid pathway of *Pantoea ananatis* on the Minimum Information about a Biosynthetic Gene cluster (MiBIG) database. It should be noted that two of the *P. bathycoeliae* genes were predicted to be pseudogenes, including an alcohol dehydrogenase and one of the two LysR transcriptional regulators, although the other LysR regulator was still functional. In the *Sodalis* sp., a potential acyl homoserine lactone gene cluster was detected, but because the cluster was located at the end of a contig the complete gene cluster could not be confirmed and will need further investigation. The genes present had 68% similarity to the gene cluster in *Sodalis praecaptivus*.

Bacterial secretion systems were also investigated in both symbionts. *Pantoea bathycoeliae* encoded for one Type 5 secretion system (T5aSS) but was predicted as a potential pseudogene due to a 40% shorter gene than its orthologs. T5aSSs consist of a single protein that also encodes for the secretion product. InterPro analyses classified the secreted domain as a pectin lyase (IPR012332), which can be considered a virulence factor. When compared to the other *Pantoea* symbiont genomes, orthologs were present only in the *Mormidea* symbiont, *Plautia stali* symbionts A, C, and F, and in the free-living species *P. dispersa* and *P. vagans*. The *Sodalis* symbiont encoded for a Type 1 secretion system (T1SS), three Type 3 secretion system (T3SS), and one T5cSS system. The T1SS can secrete a range of substrates, involved in biofilm formation, toxins, iron uptake, proteases, or lipases. The T5cSS encodes for adhesin proteins that play a role in cell attachment, and the T3SS can assist with host immune suppression and establishment in the host. The one T3SS gene cluster contained three predicted pseudogenes, and only the other two T3SSs have likely remained functional.

### 3.4 Phylogenomic placement and genome comparisons of *Pantoea bathycoeliae* within the *Pantoea* genus

**Figure 8:**
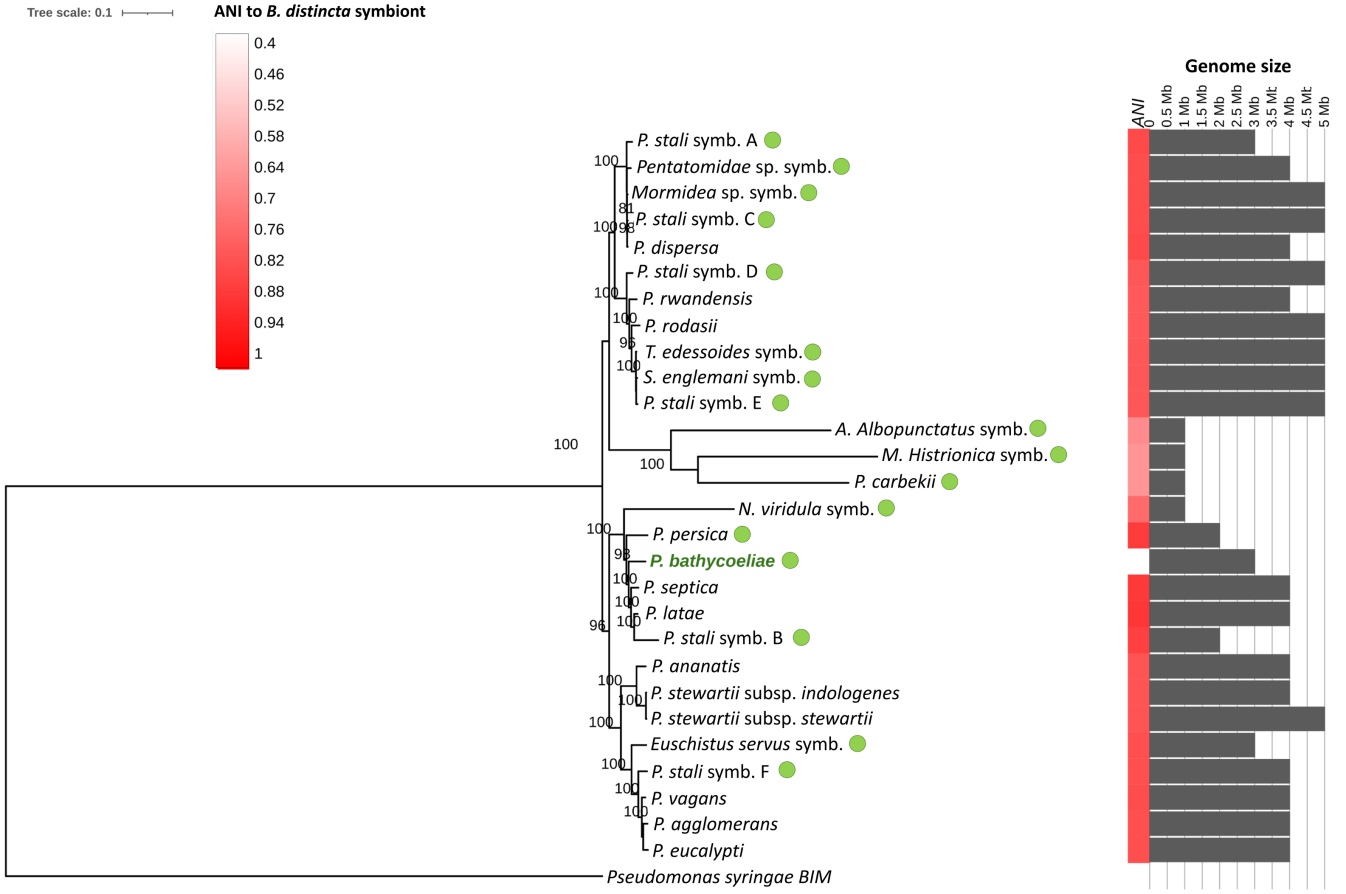
Maximum likelihood phylogenomic tree of the *B. distincta* symbiont and related *Pantoea* species and other stink bug gut symbionts (green dots), based on 358 single-copy orthologous genes (GenBank numbers of genomes are presented in Supplementary Table S1). The heatmap on the right indicates the average nucleotide identity (ANI) between the *Pantoea* symbiont and other genomes and the gray bar chart indicates genome sizes (Mb). Branch support values, based on 1,000 bootstrap replicates, are indicated on the branches.

*Pantoea bathycoeliae* had the highest genome similarity (ANI) to “*P. latae*” and *P. septica* (87% similarity). The phylogenomic analysis of 328 single-copy genes confirmed “*P. latae*” and *P. septica* as the closest related species, and the closest stink bug symbionts were *Candidatus* P. persica (from *Acrosternum arabicum*) and the *Plautia stali* symbiont B (Figure 8). This confirms that *P. bathycoeliae* is a novel *Pantoea* species. The genome size of the species is 1 Mb smaller than those of the closest free-living *Pantoea* species but 310–730 kb larger than the aforementioned *Pantoea* symbionts associated with other stink bugs. Nonetheless, the symbiotic species, *Candidatus* P. persica and *Plautia stali* symbiont B, had a highly similar number of intact protein-coding genes compared with *P. bathycoeliae* and had a pseudogene/gene ratio of 35%–45% (Supplementary Table S1).

As comprehensive genome comparisons were recently reported for the *Pantoea* gut symbionts of stink bugs (Kashkouli et al., 2021; Otero-Bravo and Sabree, 2021), only the main genome structure and any unique genes of *P. bathycoeliae* are reported in this study. The shared and unique orthologs among the *Pantoea* genomes were determined with OrthoFinder. A total of 311 genes were unique to *P. bathycoeliae*. Although many unique genes were identified, these were not linked to distinct functions in this species. The unique proteins were assigned to KEGG functional categories associated with carbohydrate metabolism, energy metabolism, nucleotide metabolism, amino acid metabolism, glycan biosynthesis and metabolism, genetic information processing, membrane transport, signal transduction, and cellular processes such as quorum sensing and biofilm formation.

### 3.5 Description of the species *Pantoea bathycoeliae*

*Pantoea bathycoeliae* (ba.ti.coe.liae L. fem. pl. suff. *Bathycoeliae*, from the *Bathycoelia distincta* stink bug species, as this bacterium was obtained from the gut of this insect).

A MAG representing this species was obtained from metagenomic sequencing of the M4 gut region of *B. distincta*. The majority of stink bug symbionts within the *Pantoea* genus are obligate symbionts and cannot be cultured *in vitro*. Despite various attempts *P. bathycoeliae* could not be isolated on nutrient agar. Phylogenetic inference of species in the *Pantoea* genus robustly supports this species as distinct in the genus and the delineation of the species is supported by a low average nucleotide identity (ANI), below 88%, with the closest related species. The genome JAUHTP000000000, available under GenBank BioProject PRJNA987703 and BioSample SAMN36078333, is the designated nomenclatural type for the species. Extracted DNA from the *B. distincta* gut, which predominantly consists of this species, is stored at the Forestry and Agricultural Biotechnology Institute, South Africa.

## 4 Discussion

The gut of the two-spotted stink bug, *Bathycoelia distincta*, has a clear compartmentalization, as seen in other *Pentatomidae*, with an obligate *Pantoea* symbiont in the M4 section and various secondary symbionts in the rest of the gut. The M4 symbiont was confirmed as a highly conserved and vertically transmitted species, based on its presence on the egg surfaces and all developmental life stages and in adults across geographic locations. Multilocus genotyping and phylogenomics confirmed this to be a distinct species, described in this study as *Pantoea bathycoeliae*. Most stink bug studies have only focused on the bacteria in the M4 gut section, but, in the current study, we highlight the facultative as well as core genera present in the rest of the gut. This also allowed the identification of a prominent secondary symbiont, *Sodalis* sp., with high functional overlap with *P. bathycoeliae*.

### 4.1 *Bathycoelia distincta* has a novel M4 gut symbiont that is vertically transferred

*Pantoea bathycoeliae* is considered a highly conserved and primary symbiont of *B. distincta*. The same *Pantoea* strain was present in the M4 gut section of all *B. distincta* stink bugs collected from five orchards in two provinces and the lab-reared population. In other stinkbug species, that were also collected from different locations, a single symbiont species was conserved in all populations (Duron and Noël, 2016; Hosokawa et al., 2016b), or minor haplotype differences were observed (Otero-Bravo and Sabree, 2018). The metabarcoding sequence approach identified 10 abundant *Pantoea* ASV sequences in *B. distincta*, of which six were classified as the *P. bathycoeliae* symbiont species, with minor sequence variations. Some of these variants likely relate to different strains of the species or different copies of the 16S rRNA gene in a single individual. However, some could also be the result of sequence errors, a caveat of Illumina sequence technologies.

A different species to the primary M4 midgut symbiont has rarely been observed in *Pentatomidae* (Hosokawa et al., 2016a, 2019). A species distinct from *P. bathycoeliae* was identified in this study (predominantly in the Limpopo 3 population) that was highly similar to *P. brenneri* and *P. vagans*, which are free-living *Pantoea* species. *P. brenneri* has been isolated from humans, while *P. vagans* was isolated from plant sources (Brady et al., 2010), making the latter a more likely source of the M4 symbiont. One prominent example of symbiont replacement is in *Plautia stali*, which were collected from different climatic regions in Japan (Hosokawa et al., 2016a). Six lineages of *Enterobacteriaceae* M4 symbionts (lineages A–F) were predominantly separated based on geographic location. Some of these lineages were free-living environmental strains and others obligate symbionts. In *B. distincta*, only one insect had a high abundance of the alternative *Pantoea* strain. This might suggest a symbiont replacement event in the Limpopo province, but follow-up collections are needed to confirm the prominence of the alternative species.

*Pantoea bathycoeliae* was present on the eggs and in the second instar nymphs, suggesting the instars acquired the symbiont by probing the egg surface. This probing behavior was observed in the current study and has also been recorded in other *Pentatomidae* species (Calizotti and Panizzi, 2014; Koga et al., 2022). In other stinkbug species, the M4 symbiont has been confirmed on the egg surface by methods such as egg surface sterilization, microscopy, and 16S rRNA sequencing (Prado et al., 2006; Kenyon et al., 2015; Kashkouli et al., 2020). A recent metabarcoding study of *N. viridula* egg microbiomes also detected a *Pantoea* symbiont on the egg surfaces but a *Sodalis* sp. was also present (Geerinck et al., 2022). Similarly, a *Sodalis* sp. was also present on the eggs of *B. distincta*; however, the vertical transfer of this additional symbiont is a new observation from our study. Whether this transfer is also through egg smearing or via the ovaries needs to be confirmed since *Sodalis* spp. can be transmitted transovarially (Kaiwa et al., 2010).

The phylogenetic and phylogenomic analyses from this study suggest that *P. bathycoeliae* is a novel species. The species was, therefore, named following the rules of the SeqCode (Whitman et al., 2022). The phylogenetic placement of the species suggests that it is closest to two free-living *Pantoea* species (*P. septica* and “*P. latae*”). *Pantoea septica* has predominantly been isolated from humans (Brady et al., 2010; Nadarasah and Stavrinides, 2014) with a single report from olive oil (Pizzolante et al., 2018), while “*P. latae*” was obtained from the rhizosphere of a cycad (*Zamia floridana*) but has rarely been reported since. The plant-associated species would be a more plausible ancestor of the *B. distincta* symbiont, but the closest relative to this species is more likely still unsampled.

### 4.2 *Pantoea bathycoeliae* genome suggests production of advantageous metabolites for its host

The genome of *P. bathycoeliae* was smaller (3.16 Mb) than those of most free-living *Pantoea* species (4.33 Mb–5.68 Mb) yet is larger than the genomes of the closely related obligate stink bug symbionts (*Candidatus* P. persica—2.85 Mb and *Plautia stali* symb B—2.43 Mb). Nonetheless, both *P. bathycoeliae* and *Candidatus P. persica* encoded for a similar number of ORFs and had a similar proportion of predicted pseudogenes (43%–46%). Genome reduction is a common trend in obligate stink bug symbionts as a result of their dependence on the host for energy and resources. A gradual adaptation has been hypothesized where larger genomes contain many pseudogenes (up to 40%) that are purged over time, while small genomes contain few pseudogenes since most have already been lost from the genome (Hosokawa et al., 2016a; Kashkouli et al., 2021; Otero-Bravo and Sabree, 2021). *Pantoea bathycoeliae* is also in this intermediate stage of genome reduction since many protein-coding genes were predicted, but 43% are potentially non-functional. It should be noted that different approaches to pseudogene identification were used in the related studies and thus direct comparisons should be made with caution.

Despite a large number of predicted pseudogenes in *P. bathycoeliae*, the functional roles predicted for the intact genes were highly similar to those of other stink bug symbionts. Metabolic pathways predominantly encoded for amino acids, vitamins, and co-factors. The species can produce more amino acids than some of the other stink bug symbionts, including all nine essential amino acids, whereas branched-chain amino acids (leucine, isoleucine, and valine) were incomplete in many other species (Kenyon et al., 2015; Otero-Bravo and Sabree, 2021). *Pantoea bathycoeliae* also encoded many of the same vitamins and co-factors as other stink bug symbionts, such as NAD, riboflavin, pyridoxal-phosphate, coenzyme A, and heme. Similar to other obligate symbionts (Kenyon et al., 2015; Otero-Bravo et al., 2018; Kashkouli et al., 2021; Otero-Bravo and Sabree, 2021), this species thus serves an important beneficial role in nutrient supplementation for *B. distincta*.

In addition to amino acid and vitamin provision, *P. bathycoeliae* likely produces a carotenoid metabolite. Carotenoids contribute to important functions in insects such as pigmentation, antioxidants, precursor of hormones, or the production of vitamin A (Heath et al., 2013). These compounds are often obtained from plant food sources but can also be produced by microbes. For example, *Benitsuchiphilus tojoi*, the symbiont of the parastrachiid stink bug *Parastrachia japonensis* has a carotenoid biosynthesis pathway (Mondal et al., 2020). Carotenoid production has also been confirmed in other free-living *Pantoea* species, such as β-carotene in *P. septica* (Pizzolante et al., 2018), and carotenoid biosynthetic gene clusters are encoded in *P. agglomerans*, *P. stewartii*, and *P. ananatis* (Kautsar et al., 2019). Biochemical assays (HPLC studies) in four stink bug species, including *Halyomorpha halys* and *Nezara antenata*, identified four carotenoid derivates (β-carotene, β-cryptoxanthin, lutein, and zeaxanthin) in these species (Maoka et al., 2021). These were suggested to have a plant origin but production by bacterial symbionts cannot be excluded. It is thus possible that *P. bathycoeliae* produces a carotenoid derivative, advantageous to its host, but functional studies are required to confirm this.

### 4.3 *Bathycoelia distincta* has a low bacterial diversity in the rest of its gut

The microbiome composition in the rest of the *B. distincta* gut (excluding the M4 section) was not highly diverse, as seen in the low alpha diversities of the populations. A similar low alpha diversity was observed in the M1–M3 gut sections of *N. viridula*, with many transient microbes and few core microbiota (Medina et al., 2018). This is also a general phenomenon in insects such as bees, aphids, and herbivorous Hemipterans (Gauthier et al., 2015; Kwong and Moran, 2016; Li et al., 2022). Interestingly, the lab-reared population did not have a lower alpha diversity than the orchard-collected samples, suggesting the lab-rearing did not reduce gut microbe diversity. In addition, many of the genera present in the native starter population of the lab population (Limpopo 2 farm) were still present in their guts, indicating the long-term maintenance of gut microbes over many generations.

The two populations from Mpumalanga had a lower gut microbiome diversity than the other populations and little variation between the orchards. This high similarity could be due to similar pest management practices since the two orchards are managed by the same company. For example, a specific pesticide regime could influence the bacterial composition and diversity of the populations. Pesticide treatments in honey bee populations have shown modifications of the gut microbial community by some neurotoxic insecticides (Rouzé et al., 2019; Zhu et al., 2020), while others had no effect on the microbiome (Raymann et al., 2018; Wintermantel et al., 2018). Due to limited sampling sites in the two different provinces, other confounding factors such as climatic differences between the provinces cannot be excluded.

### 4.4 Three core genera were observed in *Bathycoelia distincta*

Only three core genera were detected in the rest of the gut in all populations (>75% of individuals), *Pantoea*, *Sodalis,* and *Serratia*. Some genera were more region-specific such as *Yokenella* and the alternative *Pantoea* strain that were predominantly in the Limpopo and lab populations, while *Acinetobacter* and others were only in Mpumalanga. This suggests that many of the bacterial species are transient with no strong selection and could even have been obtained from food sources. Interestingly, *Yokenella* was one of the few non-transient genera in *N. viridula* and might thus be a more common gut microbe of stink bugs (Medina et al., 2018). Other transient genera detected in *N. viridula*, which were also present in *P. bathycoeliae*, were *Bacillus* and *Staphylococcus*.

*Serratia* species were present in most of the *B. distincta* individuals but were only abundant in some individuals of the lab population. Some *Serratia* species can be beneficial to their host, such as *S. symbiotica* in aphids, that provides additional vitamins or protection against parasites (Oliver et al., 2014; Renoz et al., 2022). Yet, *Serratia marcescens* is considered an insect pathogen (Slatten and Larson, 1967). This pathogen was detected in *N. viridula* midguts and was more abundant in lab populations compared to wild samples collected from soya fields (Medina et al., 2022). Further sequencing would be required to confirm the species present in *B. distincta*.

The most prominent secondary gut symbiont was *Sodalis* as it was present in all populations and highly abundant in some individuals. This species could not be clearly distinguished, phylogenetically, from other *Sodalis* stink bug symbionts and likely the same species can infect various stink bug species. It was most closely related to the *Sodalis* strain collected from the stink bug species, *Antestiopsis thunbergia*, where it was present in the gut and other tissues of about half of the individuals investigated (Matsuura et al., 2014). *Sodalis* symbionts were first reported in tsetse flies but have since been observed in weevils, psyllids, aphids, mealybugs, and a diversity of stink bugs (Hosokawa et al., 2015; Ghosh et al., 2020). However, in most stink bug species, *Sodalis* infections are rare (<5%), except for the Urostylididae family which shows very high infection rates (60%–100%) (Kaiwa et al., 2010, 2014; Hosokawa et al., 2015). The high prevalence of *Sodalis* in *B. distincta* is thus a rare phenomenon and suggests a strong selection for the symbiont in this stink bug species.

Despite the presence of both species in the M4 and the other gut compartments, *Sodalis* sp. cannot outcompete *P. bathycoeliae* in the M4 section, whereas in the rest of the gut, the *Sodalis* sp. is sometimes in much higher proportions. This might be attributed to a physical constriction in the M4 compartment, as well as physical traits of the bacterium (such as motility), as seen in *Rhiptortus pedestris* and its *Burkholderia* symbiont (Ohbayashi et al., 2015). With competitive infection assays other bacteria could also colonize the M4 region but when co-inoculated with the *Burkholderia* symbiont, the latter outcompeted them (Itoh et al., 2019). The location of *Sodalis* could also have an impact since it can be localized intracellularly in gut cells (Ghosh et al., 2020) and in this manner occupy a different niche.

The *Sodalis* sp. has some traits that might allow it to compete with other bacteria, in the rest of the gut. It encodes for a Type 5c secretion system and three copies of the type 3 secretion system. The former plays a role in cellular adhesion, which might be advantageous for the bacterium to attach and colonize the host gut (Łyskowski et al., 2011). T3SSs have generally been linked to infection and establishment advantages for bacteria in insect hosts (Dale and Moran, 2006). More specifically, *Sodalis glossinidius* encodes for two T3SSs that have been suggested to contribute to its invasion and establishment in tsetse flies (Toh et al., 2006). Likely these nanoweapons contribute to the establishment of *Sodalis* sp. in *B. distincta*, yet in the M4 compartment, other unknown factors prevent this bacterium from establishing itself.

The maintenance of both *P. bathycoeliae* and the *Sodalis* sp. in the gut is likely due to their highly similar metabolic capacities and some unique metabolites that can complement each other. Both species encode for most known amino acids and many vitamins but *P. bathycoeliae* has more complete pathways for at least three amino acids. Conversely, only the *Sodalis* sp. produces proline, thiamine, and menaquinone. Thiamine production was conserved in the *Parastrachia japonensis* symbiont and is likely important for stinkbugs (Mondal et al., 2020). Although the two species had similar CAZymes and peptidases, the unique families and higher numbers of specific families in each likely contribute to different capacities of nutrient metabolism of importance to the host. The recruitment and conservation of an additional symbiont has been observed in various aphid species, where the primary symbiont lost some metabolic capacities that could be complemented by another facultative symbiont, which eventually invades the symbiotic organ (Renoz et al., 2022; Manzano-Marín et al., 2023). It could be that the *Pantoea* and *Sodalis* species in *B. distincta* are also in this process of symbiont complementation and establishment of a second important gut symbiont.

## 5 Conclusion

In this study, we identified a unique *Pantoea* species maintained in the M4 midgut section of *B. distincta*. This is in correspondence with other *Pentatomidae*, all of which maintain a unique *Pantoea* species. An additional vertically transferred secondary symbiont, *Sodalis* sp., was prominent in the rest of the gut. Other than these two abundant symbionts, most bacteria in the rest of the gut were likely obtained from the environment and were more variable. High-quality MAGs were produced for both core symbionts and large metabolic overlaps were observed in their genomes. *Sodalis* is likely maintained in *B. distincta* outside the midgut crypts for additional beneficial functions not provided by *P. bathycoeliae*. This study contributed to our understanding of the composition of *B. distincta* microbial symbionts, which is essential for the effective control of these pests. This information could contribute in future to the development of alternative biocontrol measures such as eliminating essential symbionts or performing symbiont replacements, as has been explored in other stink bug species (Sassera et al., 2013; Taylor et al., 2017; Gonella et al., 2022).

## Data availability statement

The datasets presented in this study can be found in online repositories. The names of the repository/repositories and accession number(s) can be found at: https://www.ncbi.nlm.nih.gov/genbank/, BioProject PRJNA972719 and PRJNA987703.

## Ethics statement

The manuscript presents research on animals that do not require ethical approval for their study.

## Author contributions

AF: Conceptualization, Data curation, Formal analysis, Investigation, Project administration, Writing – original draft, Writing – review & editing. SV: Data curation, Methodology, Writing – original draft, Writing – review & editing. BS: Conceptualization, Writing – original draft, Writing – review & editing. GF: Conceptualization, Funding acquisition, Methodology, Resources, Writing – original draft, Writing – review & editing.

## Footnotes

^1^ https://www.arb-silva.de

^2^ http://microbial-genomes.org
