# Supplementary material for: Pantoea bathycoeliae sp. nov and Sodalis sp. are core gut microbiome symbionts of the two-spotted stink bug
Source: Front Microbiol. 2023 Nov 30;14:1284397. doi: 10.3389/fmicb.2023.1284397 (PMC10720322; doi:10.3389/fmicb.2023.1284397)
Supplement: Supplementary file 3 [file Data_Sheet_1.docx]

Supplementary Material

# Supplementary Figures and Tables

## Supplementary Figures


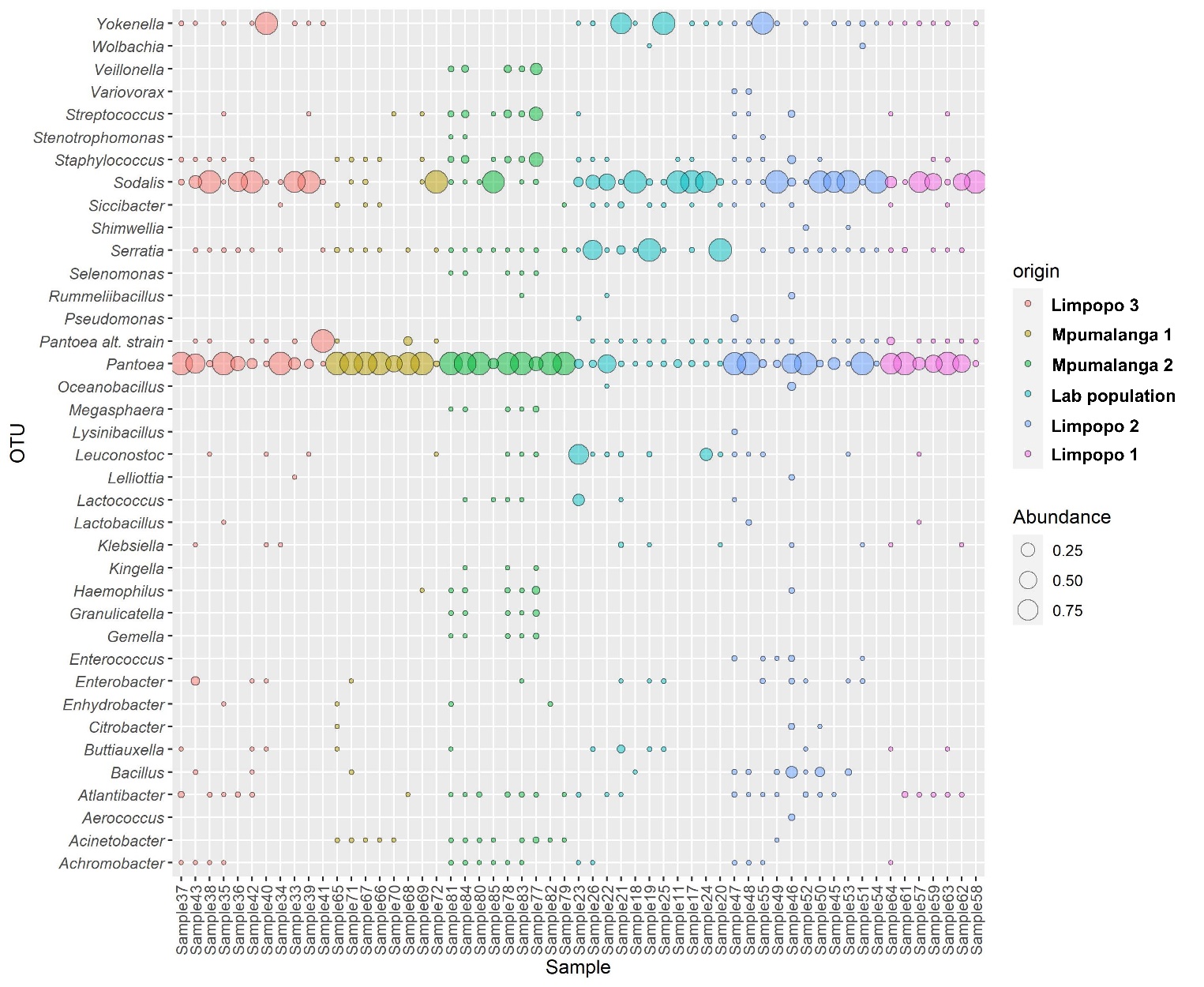


**Supplementary Figure 1.** Bubble plot based on the relative abundance of the genera in the Rest_gut compartment of each of the samples. Samples are ordered and coloured according to origin and bubble sizes indicate the relative abundance of genera in a sample. The primary *Pantoea* symbiont is indicated separate from additional *Pantoea* strains detected.


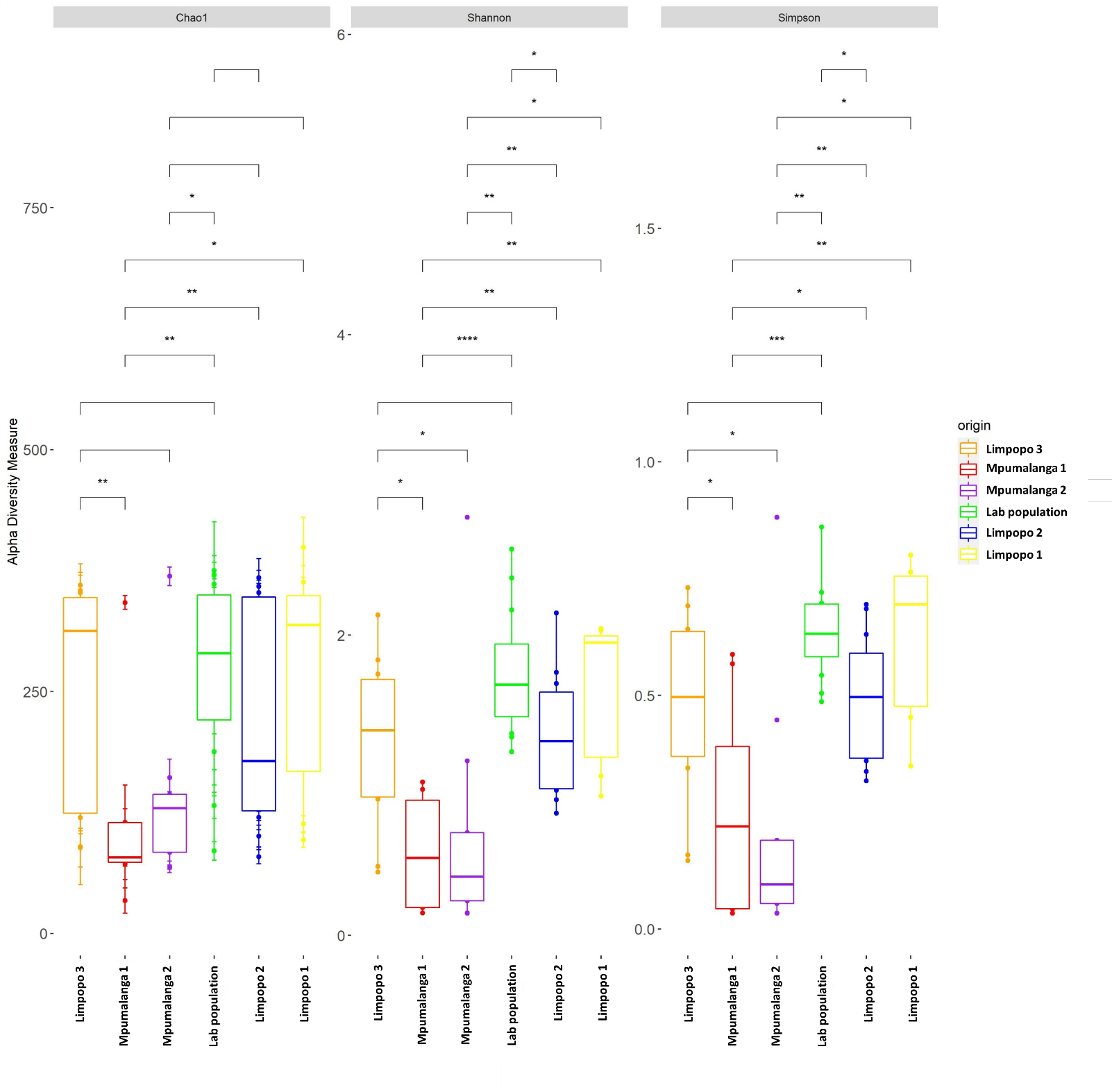


**Supplementary Figure 2**. Alpha diversity comparisons of the Rest_gut dataset between all populations investigated in this study. The Chao1, Shannon and Simpson diversity indices were considered and significant differences are indicated by asterisks (Pairwise Wilcoxon Rank Sum Tests and BH FDR testing, ****<0.0001,***< 0.001, **<, 0.01, , *<0.05).


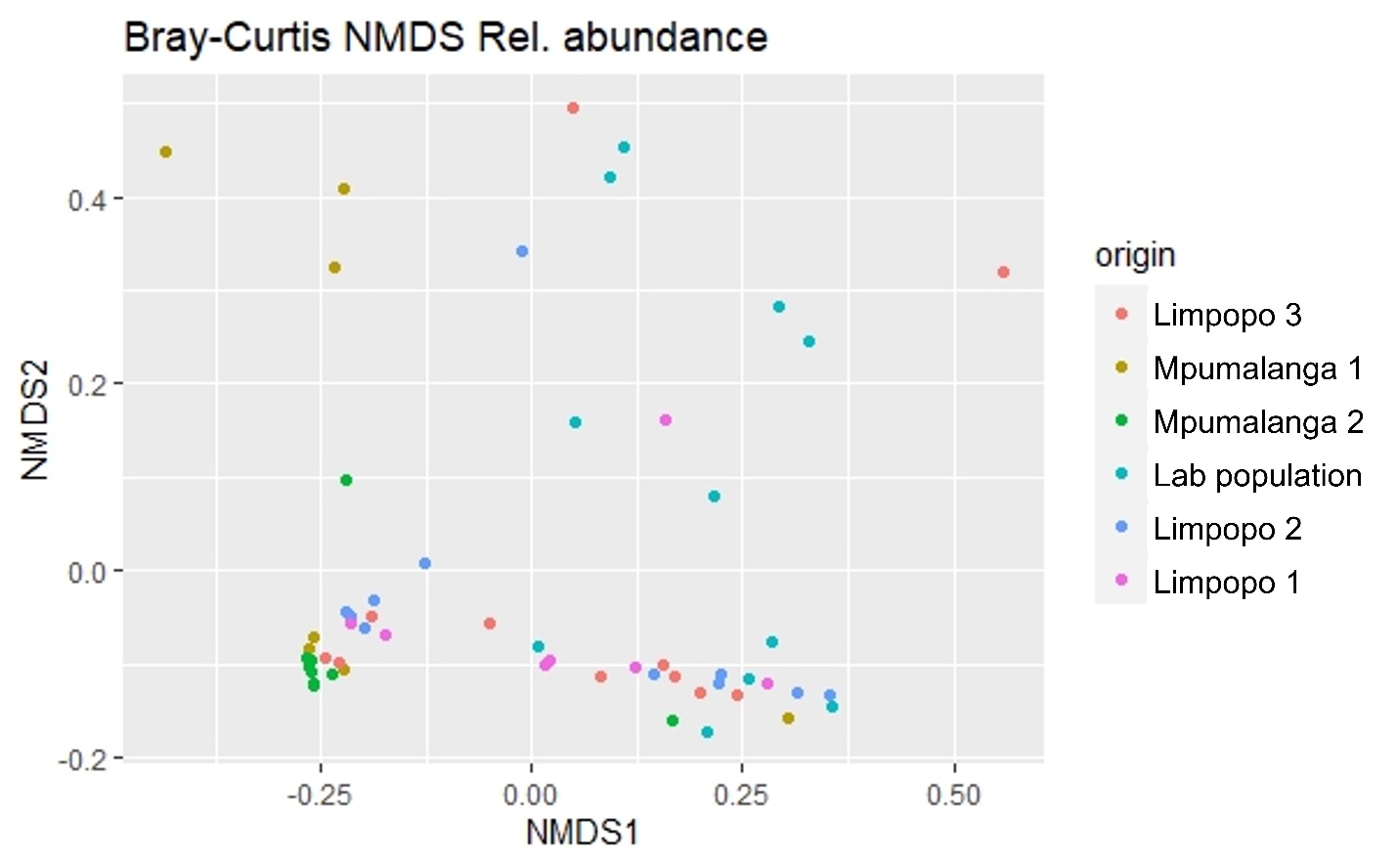


**Supplementary Figure 3**. NMDS plot, based on Bray-Curtis dissimilarities between samples. Datapoints are coloured according to geographic origin.
